# In Situ Release of Ulvan from Crosslinked Ulvan/Chitosan Complex Films and Their Evaluation as Wound Dressings

**DOI:** 10.3390/polym14245382

**Published:** 2022-12-08

**Authors:** Trong-Ming Don, Chen-Han Ma, Yi-Cheng Huang

**Affiliations:** 1 Department of Chemical and Materials Engineering, Tamkang University, No. 151 Yingzhuan Rd., New Taipei City 251301, Taiwan; 2 Department of Food Science, National Taiwan Ocean University, No. 2 Beining Rd., Keelung City 20224, Taiwan

**Keywords:** ulvan, chitosan, wound dressing, antioxidant, anti-inflammatory, wound healing

## Abstract

When a wound forms due to any injuries, it should be covered with a functional wound dressing for accelerating wound healing and reducing infection. In this study, crosslinked ulvan/chitosan complex films were prepared with or without the addition of glycerol and chlorophyll, and their wound healing properties were evaluated for potential application in wound dressing. The results showed that the tensile strength and elongation at break of the prepared ulvan/chitosan complex films were 2.23−2.48 MPa and 83.8−108.5%, respectively. Moreover, their water vapor transmission rates (WVTRs) were in the range of 1791−2029 g/m^2^-day, providing suitable environment for wound healing. Particularly, these complex films could release ulvan in situ in a short time, and the film with chlorophyll added had the highest release rate, reaching 62.8% after 20 min of releasing. In vitro studies showed that they were biocompatible toward NIH 3T3 and HaCaT cells, and promoted the migration of NIH 3T3 cells. These complex films could protect HaCaT cells from oxidative damage and reduce the production of reactive oxygen species (ROS); the addition of chlorophyll also effectively reduced the inflammatory response induced by LPS as found in the reduction in both NO and IL-6. Animal models showed that the complex films added with glycerol and chlorophyll could promote wound healing in the early stage, while accelerating the regeneration of dermal glands and collagen production. Briefly, these ulvan/chitosan complex films had good physiochemical properties and biological activity, and could accelerate wound healing both in vitro and in vivo.

## 1. Introduction

The wound-healing process is a series of complex physiological responses, including cell migration, proliferation and changes in the extracellular matrix. The wound-healing process can be divided into four stages: hemostasis, inflammation, proliferation and remodeling [1]. At each stage, specific cells are involved, such as neutrophils and macrophages for removing foreign bodies, epithelial cells which migrate to the wound for epidermal regeneration, and fibroblasts that secrete the extracellular matrix (ECM) proteins, particularly collagen and elastin, to produce connective tissue inside the wound [2]. Inflammation, epithelialization, formation of granulation, angiogenesis, wound contraction and extracellular-matrix reorganization during wound healing all occur at the same time [1,3]. To reduce wound infection and accelerate wound healing, functional wound dressings which provide a suitable warm, moist environment are widely applied for promoting rapid healing [4,5].

Ulvan (U), a sulfated polysaccharide extracted from green algae, is mainly composed of rhamnose, xylose, glucuronic acid and iduronic acid [6]. It is known that polysaccharides that are rich in rhamnose have the ability of anti-inflammation, reducing bacterial adhesion, preventing UV damage, as well as inducing collagen biosynthesis and cell proliferation [7]. In particular, the unusual carbohydrates in the backbone structure of ulvan, such as sulfated rhamnose and iduronic acid which are similar in structure to mammalian glycosaminoglycans (GAG) found in ECM of human skin [8], can enhance wound healing activities [9]. Therefore, ulvan is considered a valuable biomaterial for the development of wound-dressing materials. It has been shown that ulvan-based hydrogels could exhibit a large swelling degree, high water vapor transmission rate (WVTR), and antimicrobial and antioxidant properties; therefore, they have potential as a wound-dressing biomaterial [10]. Ren et al. [11] prepared ulvan hydrogels which were loaded with human umbilical cord mesenchymal stem cells being lyophilized to powders (hUC-MSCs), and they found that these ulvan hydrogels had the ability for enhancing diabetic chronic wound healing. The ulvan/poly(ethylene oxide) (U/PEO) nanofiber patches developed by Kikionis et al. [12] for wound dressing could effectively heal skin trauma after the cryosurgical treatment of keloids. These U/PEO nanofiber patches showed significant wound healing, elimination of inflammation, restoration of biophysical parameters to normal values, and considerable decreases in hemoglobin concentration, skin texture and volume, while no discomfort or adverse reaction was observed.

Previously, our group extracted ulvan from *Ulva lactuca* to prepare ulvan/chitosan (U/CS) complex films by ionic crosslinking [13]. The U/CS complex films exhibited significant antioxidant and whitening activities mainly due to the presence of ulvan. Moreover, the addition of chlorophyll to films could increase the release of ulvan from complex films and enhance their bioactivities, showing a synergism effect. In addition, the U/CS complex films presented cell selectivity, which were biocompatible to normal cells but toxic to melanoma cancer cells. This work is a follow-up study of ulvan/chitosan complex films, yet focusing on their potential as wound dressings. In addition to the analysis of ulvan release and material properties, in vitro cellular studies were performed to assess cell migration, and antioxidant and anti-inflammatory capabilities. Finally, the animal model was carried out to evaluate the efficacy of ulvan/chitosan complex films with the addition of glycerol and chlorophyll as functional wound dressings to accelerate wound healing.

## 2. Materials and Methods

### 2.1. Materials

*Ulva lactuca* was obtained from Taiwan Fertilizer Co. (Taipei, Taiwan). Chitosan (CS; molecular weight of 192 kDa, degree of deacetylation of ca. 85%) was supplied by Sigma Chemical Co. (St. Louis, MO, USA). Murine RAW 264.7 (murine macrophage), NIH 3T3 (mouse fibroblast), and HaCaT cells (human keratinocyte cells) were provided by the Bioresource Collection and Research Center (BCRC), Hsinchu, Taiwan. The culture medium for NIH 3T3 and HaCaT cells was DMEM/High glucose medium (Gibco^®^, Thermo Fisher Scientific Inc., Waltham, MA, USA) supplemented with 10% fetal bovine serum (FBS), 3.7 g of sodium bicarbonate and antibiotics (100 U/mL Penicillin and 100 μg/mL Streptomycin), whereas the medium for culturing RAW 264.7 was also DMEM/High glucose medium yet supplemented with 10% FBS, 2.5 g of sodium bicarbonate, 3.7 g of HEPES and antibiotics (100 U/mL of Penicillin and 100 μg/mL of Streptomycin). All other chemicals were of reagent grade and purchased from Sigma Chemical Co., unless stated otherwise.

### 2.2. Extraction of Ulvan Polysaccharides

For ulvan extraction, 100 g of air-dried alga was suspended in 2.0 L of water and autoclaved for 15 min at 121 °C. After cooling, the aqueous solution was separated by successive filtration and concentrated under a reduced pressure. Subsequently, ulvan was precipitated by 95% (*v*/*v*) ethanol, collected by centrifugation (9600 rpm for 20 min at 4 °C), and then obtained by lyophilization. In our previous study [13], it was shown that the yield of ulvan extracted from *U. lactuca* was 21.76 ± 1.60 wt%. The total sugar content and the sulfate group of ulvan extract were 41.95 ± 1.80 wt% and 16.25 ± 1.19 wt%, respectively. It had a uronic acid content of 13.75 ± 0.01 wt%, whereas rhamnose was the main sugar unit (1024.4 ± 6.6 μmol/g) along with three other sugars including xylose (229.1 ± 6.7 μmol/g), glucose (129.3 ± 0.9 μmol/g), and mannose (78.5 ± 0.5 μmol/g). The molecular weight of ulvan was 2200 kDa measured by the method also described in the research of Don et al. [13], in which dextran was used as the standard.

### 2.3. Preparation of Ulvan/Chitosan Complex Films

CS was dissolved in 1% (*v*/*v*) acetic acid solution, whereas U and tripolyphosphate (T) were dissolved in deionized water (ddH_2_O). The U, CS, and T solutions were mixed homogeneously, and the mixed solution was cast onto a plastic mold (7.6 cm in diameter). The weight ratio of U, CS, and T was fixed at 20/75/5, and the prepared film was denoted as U/CS/T. Additionally, glycerol (G) and chlorophyll (Chl) were added separately to the mixed solution to prepare U/CS/T-G and U/CS/T-GChl films. The glycerol addition was added at 10 wt% based on the CS weight. The chlorophyll added was 0.2 wt% based on the weight of the U/CS/T film.

### 2.4. Characterizations of Ulvan/Chitosan Complex Films

For evaluating tensile mechanical properties, the complex films were cut into a rectangular shape (8 cm × 2 cm) and their ultimate tensile strength (MPa) and elongation at break (%) were then measured using a texture analyzer (Model CT3, Brookfield, Middleboro, MA, USA) with a moving speed of 1.0 mm/s [14].

To evaluate moisture content at equilibrium, the sample film was first incubated in an environmental chamber at a constant temperature of 25 °C and a relative humidity (R.H.) of 75% for one week. The film was taken out and weighed (*W_eq_*). Subsequently, the film was placed in an oven at 105 °C for 24 h and then weighed again (*W_d_*).

$$\mathrm{Equilibrium}\;\mathrm{moisture}\;\mathrm{content}\;(\%)=\frac{W_{eq}\;{-\;W}_{d}}{W_{d}}\;\times \;100\;(\%)$$

To evaluate swelling degree, the sample film (2 cm × 2 cm) was immersed in ddH_2_O and removed at several specific time points.

$$\mathrm{Swelling}\;\mathrm{degree}\;(\%)=\frac{W_{t}\;{-\;W}_{d}}{W_{d}}\;\times \;100\;(\%)$$

where *W_d_* (g) is the mass of dried film and *W_t_* (g) is the mass of the film after immersion in water at a specific time point.

The water vapor transmission rate (WVTR) was evaluated at 37 °C and 50% R.H. by the method according to Jiang et al. [15].

$$\mathrm{WVTR}=\frac{W_{o}{\;-\;W}_{t}}{t\;\times \;A}\;(g\;m^{-2}{\mathrm{day}}^{-1})$$

where WVTR is expressed in g m^−2^day^−1^, *W_o_* (g) is the initial mass of the device, *W_t_* (g) is the final mass of the device, *t* (day) is the time of weighing, and *A* (m^2^) is the surface area of the test tube mouth covered with the sample film (I.D. = 4.0 cm).

### 2.5. In Vitro Release of Ulvan in Distilled Water

The complex films (2 cm × 2 cm) were immersed in 5 mL of ddH_2_O. Each time, 1 mL of supernatant was withdrawn at one specific time point and replaced with an equal volume of ddH_2_O. Then, the amount of ulvan released in supernatant was determined by measuring its sulfate content, which was quantified by the BaCl_2_-gelatin turbidity method, with sodium sulfate applied as a standard [16].

### 2.6. Antioxidant Activities

The samples analyzed included the CS, U/CS/T, U/CS/T-G, and U/CS/T-GChl films before and after 30 min immersion in ddH_2_O, and their respective supernatants. Four indicators of antioxidant activities were evaluated, including 1,1-diphenyl-2-picrylhydrazyl (DPPH) scavenging ability [17], superoxide radical scavenging ability [18], ferrous ion chelating ability [19], and reducing power [20]. The details of the experimental methods on the antioxidant abilities are shown in Supplementary Materials.

### 2.7. Biocompatibility Test

The cell viability toward the CS, U/CS/T, U/CS/T-G, and U/CS/T-GChl films as well as to their released media, which were prepared by immersing the complex films (1 × 1 cm) in the DMEM/High glucose medium for 24 h, was measured by MTT assay according to the manufacturer’s procedure. Briefly, HaCaT or NIH 3T3 cells with a density of 5 × 10^4^ cells/well were seeded onto a 24-well plate for 24 h of attachment. Afterward, the film was added into the well or the culturing medium was replaced with the release medium. The cell culture was continued for 1 day. The film along with the medium were then removed, and the well was added with 1 mL of MTT (0.05%, *w*/*v*). After another 4 h incubation at 37 °C, 1 mL of dimethyl sulfoxide (DMSO) was added to dissolve the purple product. The absorbances at 570 and 630 nm were then measured using a microplate spectrophotometer (SpectraMax 340PC384, Molecular Devices, Sunnyvale, CA, USA).

$$\mathrm{Cell}\;\mathrm{viability}\;(\%)=\frac{{(\mathrm{OD}}_{570-\mathrm{Sample}\;}{-\;\mathrm{OD}}_{630-\mathrm{Sample}})}{{(\mathrm{OD}}_{570-\mathrm{Control}\;}{-\;\mathrm{OD}}_{630-\mathrm{Control}})}\;\times \;100$$

### 2.8. Scratch Assay

The NIH3T3 cells were seeded in a 24-well plate at a density of 3 × 10^4^ cells per well and maintained at 37 °C and 5% CO_2_ for 24 h to allow for cell adhesion to form a confluent monolayer. Scratch was created using a 200 μL pipette tip on the confluent monolayer of cells. The culture medium along with suspended cells were removed immediately and replaced with the release medium from each complex film. After continuous culturing for 8 and 24 h, healing of scratched wound was observed by a microscope (Olympus, CKX41) and analyzed by ImageJ. The percentage of wound closure was calculated as follows:

$$\mathrm{Wound}\;\mathrm{closure}\;(\%)=\frac{{(\;A}_{t}-{\;A}_{o})}{A_{o}}\;\times \;100$$

where A_t_ is the wound area at one specified time point, A_o_ the original wound area.

### 2.9. Effect of Ulvan/Chitosan Complex Films on H_2_O_2_-Treated Cells

To determine a suitable concentration for the H_2_O_2_-induced injury model in vitro, the viability of HaCaT cells exposed to a series of H_2_O_2_ solution was first measured. HaCaT cells at a density of 5 × 10^4^ cells/well were seeded onto a 24-well plate for 24 h of attachment. The medium was then removed, and a DMEM/high glucose medium containing a specific concentration of H_2_O_2_ (100, 200, 400, 600, or 800 μM) was added. The cell viability was then examined by using the MTT assay after 3, 4, and 6 h of exposure to H_2_O_2_ for screening the optimal concentration of H_2_O_2_ for the test of the cell oxidative damage model. The viability of HaCaT cells was found to be dose-dependent and decreased with the increasing H_2_O_2_ concentration, as shown in Supplementary Figure S1. When the H_2_O_2_ concentration was 200 μM, cell viability at 3 h, 4 h, and 6 h as compared to the control were 55.7%, 50.8%, and 39.6%, respectively. When the H_2_O_2_ concentration was higher than 200 μM, the cell viability was less than 31% after 6 h of culturing. According to Zhang et al. [21], the suitable concentration for the H_2_O_2_-induced injury model should be that the cell viability in this concentration was approximately at 50%. Thus, H_2_O_2_ of 200 μM was chosen as the optimal damage condition for the following experiment.

HaCaT cells at the same density of 5 × 10^4^ cells/well were first seeded onto a 24-well plate for 24 h of attachment. The medium was then replaced with the release medium from each complex film, and continued to co-culture with HaCaT cells for 18 h. Subsequently, 200 μM of H_2_O_2_ was added to make the cells subjected to oxidative stress. After culturing for 3 and 6 h, the cell viability was detected by the MTT assay. The untreated cells served as the control and their viability was taken as 100%. The effect of complex films on H_2_O_2_-treated cells was expressed as the relative percentage of cell viability as compared to the control.

### 2.10. Determination of Immunomodulatory Activity

#### 2.10.1. Measurement of ROS Generated by RAW 264.7 Cells

The intracellular ROS generated by RAW 264.7 cells was determined using a 2′,7′-dichlorofluorescin diacetate (DCFH-DA) assay. The DCFH-DA enters the cell where it reacts with ROS to form a highly fluorescent compound of dichlorofluorescein (DCF). Briefly, RAW 264.7 cells (2 × 10^5^ cells/well) were seeded onto a 6-well plate and allowed to attach for 24 h before treatment. The culturing medium was then replaced with the release medium from each ulvan/chitosan complex film and added with LPS (1 μg/mL); the cell culture was continued for 1 more day. Finally, the medium was removed, and a fluorescent dye (20 μM DCFH-DA) was added into the well for a 30 min incubation. After washing cells with PBS, the cells were observed by inverted fluorescence microscope (CKX41, Olympus, Tokyo, Japan) and analyzed by flow cytometry (BD FACSAria, BD Biosciences, San Jose, CA, USA).

#### 2.10.2. Quantitative Analysis of NO and Cytokines

RAW 264.7 cells at a density of 3 × 10^4^ cells/well were planted in a 24-well plate for attachment, and then the release medium from each ulvan/chitosan complex film along with LPS (1 μg/mL) was added into the well for 24 h of culturing. For nitric oxide (NO) quantification, 250 μL of 1% sulfanilamide solution was added to 500 μL of supernatant and waited for 10 min in the dark before adding 250 μL of 0.1% *N*-(1-naphthyl)-ethylenediamine for another 10 min. The absorbance of the produced dye in the solution was measured at 540 nm using a spectrophotometer (SpectraMax 340PC384, Molecular Devices, San Jose, CA, USA) [22]. For interleukin-6 (IL-6) quantification, 500 μL of the supernatant was withdrawn and analyzed by using the IL-6 ELISA kit (Thermo Fisher Scientific Inc., Waltham, MA, USA). LPS was used as a positive control to stimulate the IL-6 and validate the ELISA protocol.

### 2.11. In Vivo Wound Healing Experiments

The animal study was in accordance with the Guide for the Care and Use of Laboratory Animals. The Animal Care and Use Committee of National Taiwan Ocean University approved all procedures that involved animals. The registration number of animal ethical permission is 106037. Sprague Dawley rats (230–250 g) were used for the study. After anesthetized by Zoletil with a dose of 50 mg/kg, the back was shaved with standard animal clippers, and skin was swabbed with 70% ethanol. Next, a midline incision was made in the back, and the animal was exposed with a stainless-steel retractor. A round full-thickness skin defect of 1.5 cm diameter was made on the back of the animals parallel to the vertebral column. The wound sites were marked and measured using digital calipers and averaged to determine the original wound diameter and area. The wound was then covered with a 3M^®^ Tegaderm transparent film dressing (3 M^®^ Health Care, St. Paul, MN, USA), the U/CS/T-G film, or the U/CS/T-GChl film, all at a dimension of 2.5 cm × 2.5 cm. The wound without treatment was used as the control group.

Wound closure observation was assessed by tracing the wound with a digital camera on days 0, 3, 5, 7, 10, 14, and 21 post-surgery. Wounds were delineated with transparent plates and wound areas were quantified by using ImageJ software. The wound closure percentage was determined from the following equation:

$$\mathrm{Wound}\;\mathrm{closure}\;(\%)=\frac{A_{o}-A_{N}}{A_{o}}\times 100\%$$

where A_o_ (mm^2^) is the original wound area; A_N_ (mm^2^) is the wound area at a specific time point.

For histological examinations, the wounds and their surrounding skin areas after post-operation at a specific time point were fixed with 10% formaldehyde solution, paraffin-embedded and then stained with hematoxylin and eosin (H&E) reagent. Furthermore, the formation and distribution of collagen on the wounds were analyzed by Masson’s trichrome stain.

### 2.12. Statistics Analysis

All the data were presented in the form of mean ± standard deviation (SD). Graph Pad Prism 5 was used for statistical analysis, and the data were checked through one-way ANOVA and the post-test analysis, with significant differences of * *p* < 0.05, ** *p* < 0.01, and *** *p* < 0.001.

## 3. Results and Discussion

### 3.1. Physical Properties of Ulvan/Chitosan Complex Films

The ulvan/chitosan (U/CS) complex films were formed by directly mixing ulvan and chitosan together, and enhanced by adding tripolyphosphate (T) and glycerol (G). The polyphosphate penta-anion could serve as an ionic crosslinker for the positive-charged chitosan material [23,24], whereas the glycerol with three hydroxyl groups could have strong hydrogen bonding with polysaccharides having hydroxyl, amino, and acid groups. The FTIR analysis of the prepared U/CS/T films was carried out and the spectrum was shown in Supplementary Figure S2. The complex film had all the characteristic absorption peaks of its individual components, yet slightly shifted in position, owing to their interactions. Their mechanical properties, therefore, depended on the weight ratio of U to CS and crosslinker added. The ultimate tensile strength was increased as tripolyphosphate was added [13]. After research on several films with various compositions and comparing their physicochemical properties, the weight ratio of U/CS/T was set at 20/75/5, at which the film was found to have the optimum performance, particularly having the best mechanical properties. Moreover, the addition of chlorophyll (Chl) could increase the ulvan released from the complex films. Therefore, in this study, we chose U/CS/T, U/CS/T-G, and U/CS/T-GChl, all with the same U/CS/T weight ratio of 20/75/5 for the following analysis to evaluate their potential as wound dressings. Moreover, pure CS film was prepared for comparison.

**Figure 1:**
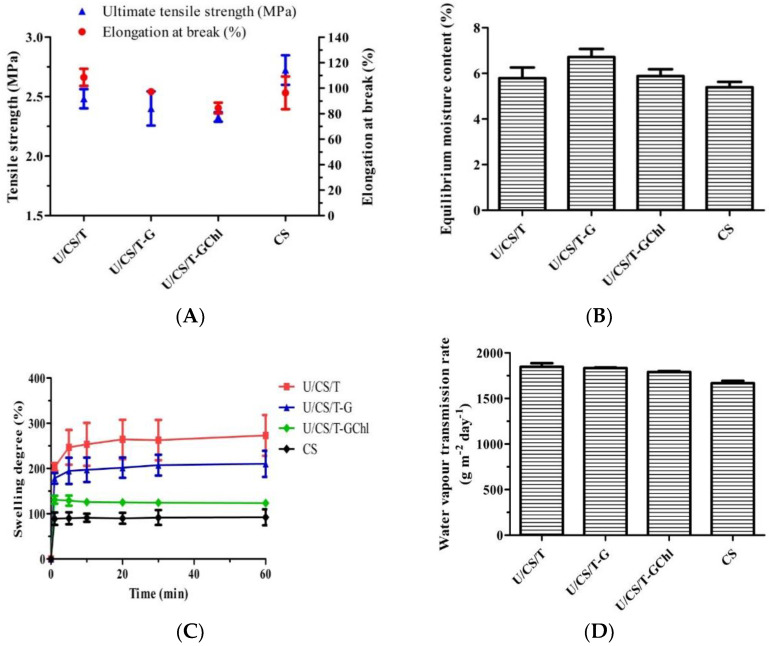
Several physical properties of the chitosan and ulvan/chitosan complex films: (**A**) ultimate tensile strength and elongation at break, (**B**) equilibrium moisture content, (**C**) swelling degree, and (**D**) water vapor transmission rate. All the complex films had the same composition of U/CS/T at 20/75/5. Data were represented by the mean ± SD, *n* = 3.

As shown in Figure 1A, among all the groups, the neat CS film had the highest ultimate tensile strength of 2.72 ± 0.10 MPa, followed by the U/CS/T film with a value of 2.48 ± 0.06 MPa. As for elongation at break, the U/CS/T film had the highest value of 108.5 ± 5.5%, followed by the U/CS/T-G film with 97.3 ± 1.4%. The U/CS/T-GChl film exhibited the lowest value of both ultimate tensile strength and elongation at break. Previous studies indicated that normal human skin exhibited tensile strengths in the range of 2.5–16 MPa and elongation at break of 35–115% [25,26]. The values of ultimate tensile strength of the prepared complex films were close to or slightly lower than 2.5 MPa, but the elongations at break were within the range, presenting potential as a wound dressing.

The equilibrium moisture content at 25 °C and 75% R.H. is shown in Figure 1B with values between 5.79% and 6.72%. The CS film had the lowest moisture content, while the U/CS/T-G film had the highest value. The likely explanation is that ulvan and glycerol are more hydrophilic than CS, making them easier to absorb moisture. Consequently, the U/CS/T-G film that had both hydrophilic ulvan and glycerol exhibited the highest moisture content. When the Chl—which is known to be hydrophobic—was further added to prepare the U/CS/T-GChl film, the moisture content decreased again, as revealed in Figure 1B.

The swelling behavior of the complex films is shown in Figure 1C. All the films swelled rapidly in the first minute, and then gradually reached their respective plateau values. After 60 min, the swelling degrees of the U/CS/T, U/CS/T-G, U/CS/T-GChl, and CS films were 273.2%, 210.4%, 123.8%, and 92.2%, respectively. The swelling degree was the lowest for the CS film, and it increased three-fold for the U/CS/T film because of the highly hydrophilic ulvan added. The interaction between the charged carboxylate and sulfate groups of ulvan and water greatly increased the swelling ability [27]. Yet, the addition of glycerol increased the crosslinking extent, thus, lowering the swelling degree. A further addition of chlorophyll endowed the complex film with a lower swelling ability, which might be due to the hydrophobicity of the chlorophyll molecule.

Effective wound dressings must be able to maintain a suitable moist environment for prolonged periods of time to prevent excessive wound dehydration and accumulation of exudate, facilitate wound re-epithelialization, and prevent scar formation [28]. Therefore, WVTR is an important indicator for wound-dressing evaluation. As shown in Figure 1D, the CS film had the lowest value, and the other ulvan/chitosan complex films had WVTR values between 1641 and 1888 g m^−2^day^−1^. The films with higher swelling degrees (U/CS/T and U/CS/T-G) exhibited higher WVTR values as well. The addition of hydrophilic components such as ulvan and glycerol could increase the WVTR value of the films [29]. A further addition of Chl (U/CS/T-GChl) lowered the WVTR as compared to the U/CS/T-G film.

### 3.2. In Vitro Ulvan Release in Distilled Water

**Figure 2:**
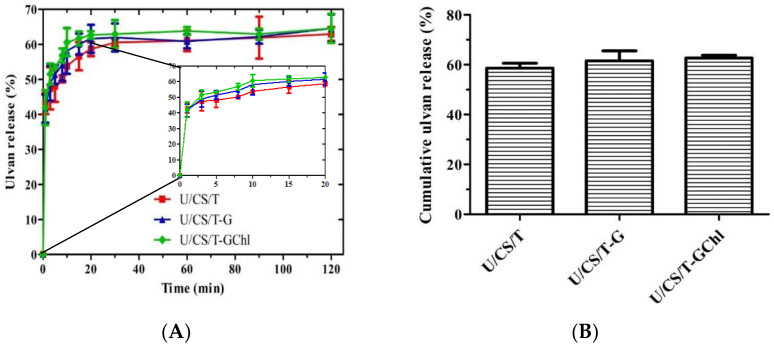
(**A**) Ulvan release percentage from various ulvan/chitosan complex films in distilled water; (**B**) Comparison of the ulvan release percentage at 20 min from various ulvan/chitosan complex films in distilled water. All the complex films had the same composition of U/CS/T at 20/75/5. Data were represented by the mean ± SD, *n* = 3.

As shown in Figure 2A, ulvan rapidly released from the complex films when immersed in distilled water in the first 5 min and then gradually increased to a plateau value at ca. 20 min. This is because ulvan is a highly hydrophilic polysaccharide. As in the enlarged view of release for 20 min in Figure 2A, the cumulative ulvan release percentages from the U/CS/T, U/CS/T-G, and U/CS/T-GChl films were 58.7%, 61.6%, and 63.3% at 20 min, respectively (Figure 2B). Interestingly, the order of ulvan release from the films was opposite to the order of the swelling degree. The addition of glycerol and Chl decreased the swelling degree but increased the amount of ulvan release. This might be due to the decreasing interaction between ulvan and chitosan when glycerol and Chl were added, resulting in the higher release of ulvan.

### 3.3. Antioxidant Activities of Ulvan/Chitosan Complex Films

**Figure 3:**
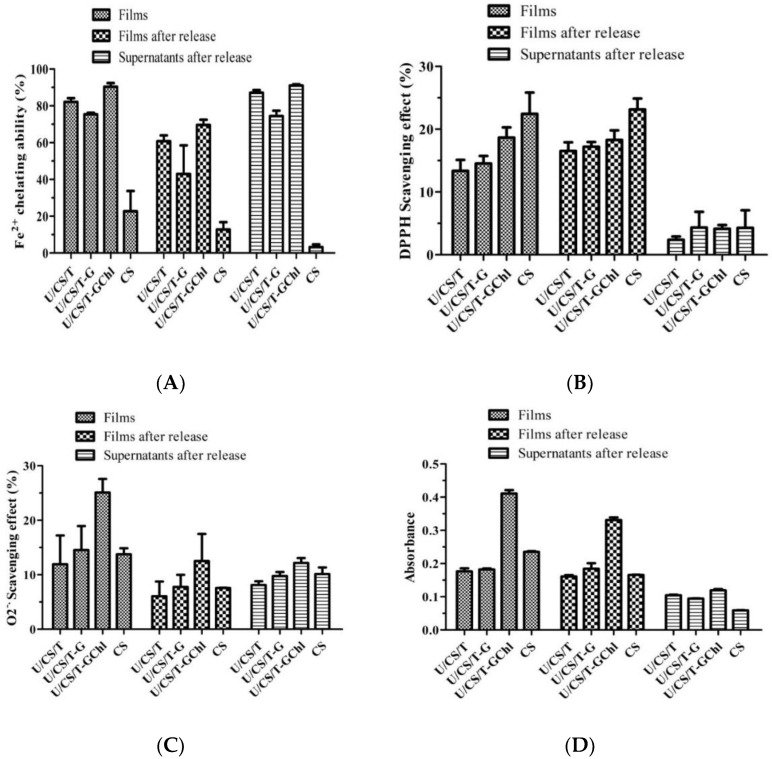
Antioxidant activities of the ulvan/chitosan complex films before and after ulvan release, and their corresponding supernatants after release (**A**) DPPH radical scavenging activity, (**B**) ferrous iron chelating ability, (**C**) superoxide anions scavenging activity, and (**D**) reducing power activity. All the complex films had the same composition of U/CS/T at 20/75/5. Data were represented by the mean ± SD, *n* = 3.

As shown in Figure 3, the antioxidant activities of complex films were investigated by DPPH and superoxide scavenging ability, ferrous iron chelating ability, and reducing ability. The results showed that the antioxidant activity of complex films came from the film itself and the released ulvan in the supernatant, and the addition of chlorophyll further increased antioxidant activity, in agreement with the previous results [13]. Figure 3A reveals that the neat CS film had the highest DPPH radical scavenging activity, and the addition of tripolyphosphate and ulvan decreased the activity significantly. As already explained by Don et al. [13], this was due to the interactions of the amino groups of chitosan with the penta-anions of polyphosphate and also the sulfate and carboxylic groups of ulvan, resulting in fewer amino groups available. Akyuz et al. [30] also found that DPPH radical scavenging activity was lower when fewer amino groups were available in the chitosan-based films. Moreover, Figure 3A shows that the further addition of hydrophobic Chl restored the DPPH radical scavenging activity. For the ferrous iron chelating ability, as shown in Figure 3B, all the ulvan/chitosan complex films including U/CS/T, U/CS/T-G, and U/CS/T-GChl exhibited extremely high ferrous iron chelating ability rather than CS film, especially in the released supernatants of these films. It is clear that ulvan is mainly responsible for the ferrous ion chelating ability, as ulvan could be released only from the ulvan/chitosan complex films (cf. Figure 2). Ulvan has been reported to have antioxidant activities, attributed to its strong hydrogen supply ability and metal chelation ability [31,32,33]. As for superoxide scavenging ability and reducing power (Figure 3C,D), the U/CS/T-GChl film had a relatively high ability as compared to other films, which might be attributed to the synergistic effect of ulvan and Chl. Cho et al. [34] indicated that the antioxidant activity of *Enteromorpha prolifera* (*E*. *prolifera*) extraction was positively correlated with the amount of Chl compounds. Chlorophyll derivatives could act as an electron acceptor to reduce DPPH and other free radicals [35]. Le Tutour et al. found that the antioxidant activity of vitamin E was enhanced by adding Chlorophyll *a* [36].

### 3.4. Biocompatibility

**Figure 4:**
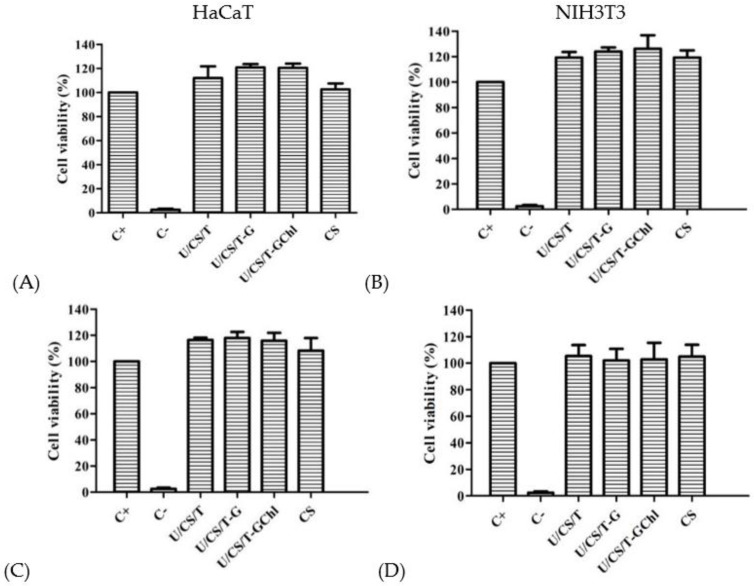
Cell viability of HaCaT cells treated with the ulvan/chitosan complex films (**A**), and their released media (**B**); cell viability of NIH 3T3 cells treated with the ulvan/chitosan complex films (**C**), and their release media (**D**). All the complex films had the same composition of U/CS/T at 20/75/5. Data were represented by the mean ± SD, *n* = 3. Positive control (C+) is culturing medium, Negative control (C−) is 10% DMSO in medium.

The ulvan/chitosan complex films and their released media obtained by immersing the complex films in culturing medium for 24 h were separately co-cultured with human keratinocytes (HaCaT) or mouse fibroblasts (NIH 3T3) to evaluate their biocompatibility. The results are shown in Figure 4. The cell viability percentages as compared to the control were all above 100%, indicating that not only the films but also their released substances, which were mainly ulvan, were biocompatible to HaCaT and NIH 3T3 cells. Ulvan extracts have been reported to exhibit no cytotoxicity to a variety of cells, such as macrophages, intestinal cells, and fibroblast cells [37]. Previous studies indicated that ulvan extracted from *Ulva lactuca* had no toxic effects on HaCaT cells [38] and L929 cells determined by MTS assay, dsDNA, and total protein analysis [39]. The ulvan nanofibrous membranes developed by Toskas et al. also presented biocompatibility by improving the adhesion and proliferation of osteoblasts [40].

### 3.5. Scratch Assay

**Figure 5:**
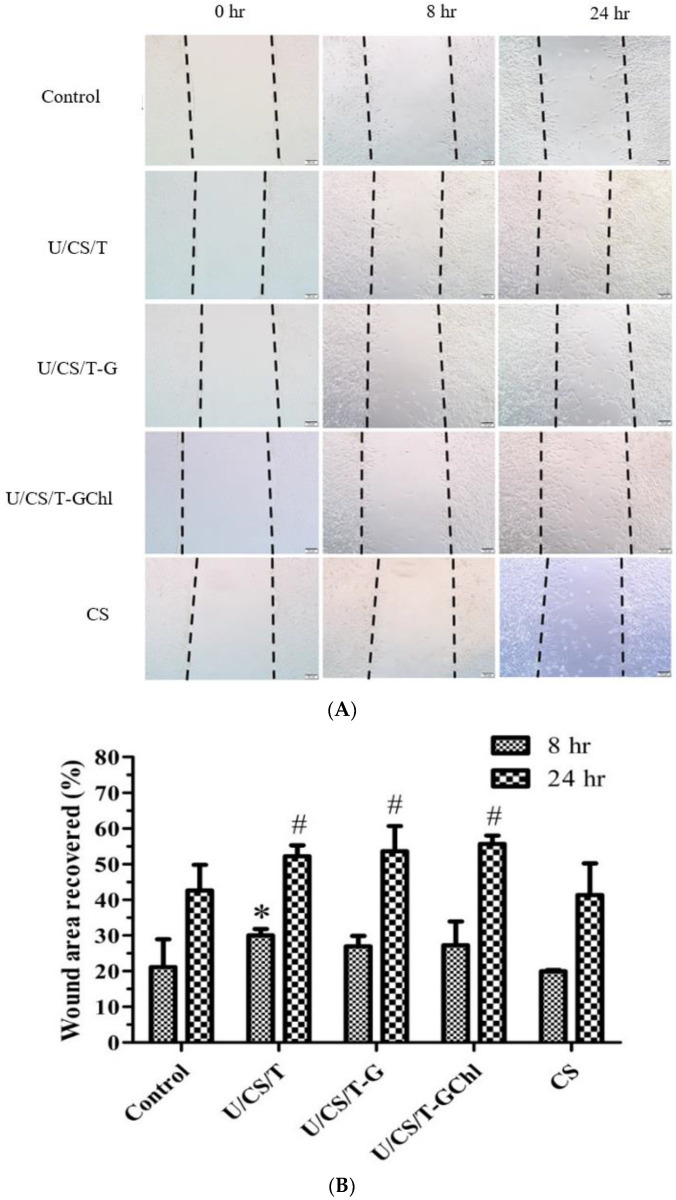
(**A**) Optical micrographs of NIH 3T3 cell migration affected by the ulvan/chitosan complex films. Scale bar = 500 μm; (**B**) Analysis of the migration ability as expressed by wound closure (%). Data represent mean ± SD from 3 separate experiments. Control is medium. * *p* < 0.05 compared with the control group at 8 h. # *p* < 0.05 compared with the control group at 24 h.

The scratch assay was adopted to evaluate the migration ability of fibroblast cells affected by the ulvan/chitosan complex films in vitro. The optical micrographs of NIH 3T3 cell migration are shown in Figure 5A, and the analysis of their migration ability, as expressed by wound closure percentage (%), is presented in Figure 5B. At 8 h, the U/CS/T film had the maximum wound area recovery with a value of 30.0%. Yet, for the other three groups, there was no statistical difference as compared with the control group. After 24 h of observation, the wound recovery of all ulvan/chitosan complex films was better than that of the neat CS film. The film that had the best performance was the U/CS/T-GChl, with 55.69% of the wound area recovered. Our experimental results thus revealed that ulvan had the ability to promote fibroblast migration, which was important during wound healing. Ulvan has a similar structure and function to glycosaminoglycans (GAGs), such as heparin and chondroitin sulfate. Heparin is frequently used for wound healing by inducing fibroblast proliferation and improving fibrinolytic function. Meanwhile, chondroitin sulfate has been reported to play an important role in the wound-healing process through influencing fibroblast adhesion [41,42]. Thus, we believe that ulvan has potential to affect fibroblast behavior to promote wound healing. In addition, the polysaccharides extracted from the *Gracilaria lemaneiformis*, a kind of red alga, have been demonstrated to promote HaCaT cell migration and proliferation by activating the PI3K/aPKC pathway [43].

### 3.6. Effect of Ulvan/Chitosan COMPLEX films on H_2_O_2_-Treated Cells

Reactive oxygen species (ROS) have been considered to play a pivotal role in the wound-healing process at various stages [44]. The excessive production of ROS leads to oxidative stress, which adversely affects wound healing and hinders new tissue formation [45]. Hydrogen peroxide (H_2_O_2_), a principal member of ROS, is critical in various physiological processes, such as cell growth, immune response, and senescence [46]. Therefore, we assessed the effects of ulvan/chitosan complex films on the viability of HaCaT cells after being treated with H_2_O_2_ to evaluate its potential as a wound-dressing material.

**Figure 6:**
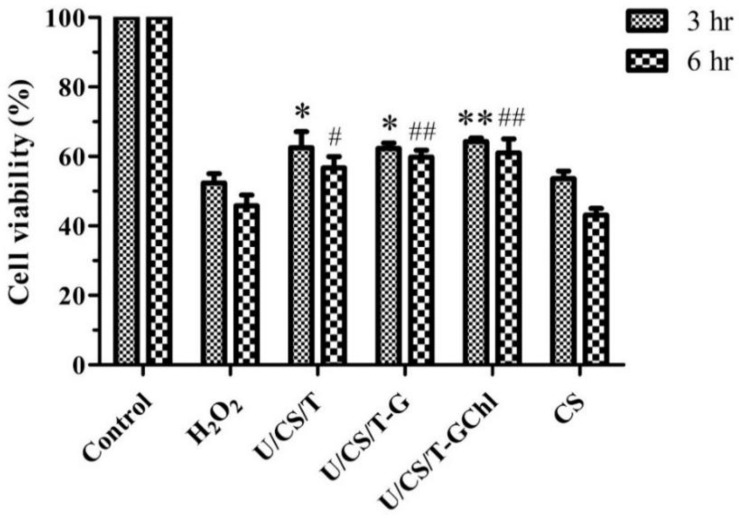
HaCaT cells were pre-cultured with the released media from the ulvan/chitosan complex films for 18 h prior to exposure to H_2_O_2_ (200 μM). Control is the medium without H_2_O_2_. * *p* < 0.05, ** *p* < 0.01 compared with the 3-hour H_2_O_2_ group. # *p* < 0.05, ## *p* < 0.01 compared with the 6-hour H_2_O_2_ group.

As shown in Figure 6, pretreatment with the ulvan/chitosan complex films before H_2_O_2_ exposure improved the cell viability. Moreover, the U/CS/T-GChl group exhibited the highest cell viability with values of 64.3% and 61.0% at 3 h and 6 h, respectively. However, the cell viability of the CS group was close to that of the H_2_O_2_ treatment group, indicating that the CS film had no protective effect on the cells subjected to H_2_O_2_ damage. The likely explanation is that the protective effect of the ulvan/chitosan complex films from H_2_O_2_ might be related to ulvan, which was released during the immersion of complex films in the culturing medium (cf. Figure 2). Compared to the ulvan/chitosan complex films, the neat CS film clearly had no ulvan to be released. In addition, research by Cai et al. indicated that the polysaccharide extracted from the green alga *Ulva prolifera* could also inhibit the damage of cells induced by H_2_O_2_, demonstrating its ability of eliminating free radicals and reducing the lipid peroxidation [47]. Antioxidative polysaccharides can enhance cell viability against oxidation-induced cell death, thereby protecting living cells from oxidative damage caused by free radicals [48].

### 3.7. Immunomodulatory Activities

**Figure 7:**
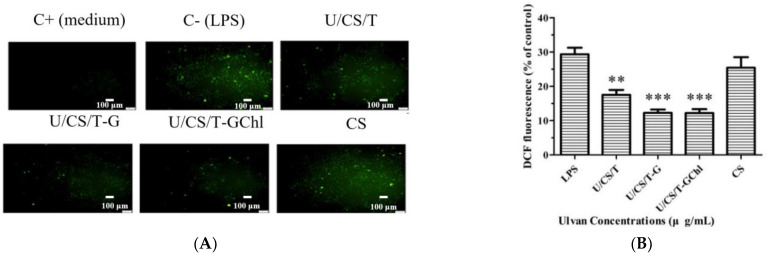

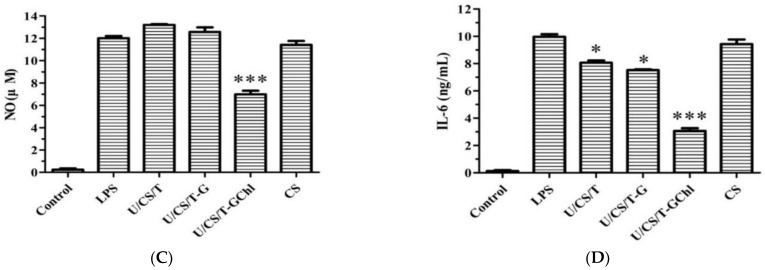
(**A**) Inverted fluorescence microscopic images, (**B**) flow cytometry analysis of intracellular production of ROS, and secretion of (**C**) NO and (**D**) IL-6 in LPS-stimulated RAW264.7 cells cultured in various released media of the ulvan/chitosan complex films. Data represent mean ± SD from 3 separate. Control is the medium without LPS. * *p* < 0.05, ** *p* < 0.01, *** *p* < 0.001 compared with the control group.

It is known that an excess amount of ROS is detrimental to wound healing and hinders new tissue formation [49]. The intracellular ROS was selected to be an effective biomarker to reflect the immunomodulatory effect of the ulvan/chitosan complex films on RAW 264.7 cells. ROS have been shown to be involved in the synthesis of a series of inflammatory factors or to enhance the ability of cells to phagocytose, killing bacteria and other foreign substances [50]. Thus, LPS was used to induce RAW264.7 cells to differentiate into an inflammatory state, resulting in ROS generation and oxidative stress, and then the intracellular ROS were detected by inverted fluorescence microscopy and flow cytometry. The results are shown in Figure 7A,B. The ulvan/chitosan complex films displayed a significant scavenging effect on ROS. In addition, opposite trends were observed for the ROS content and ulvan release (cf. Figure 2). The more the ulvan released, the less the ROS produced. Among them, the U/CS/T-G and U/CS/T-GChl films showed the best scavenging ability with fluorescence of 12.3% and 12.2%, respectively. The fluorescence of the CS film was 25.4%, which was not statistically different from that in the LPS group, indicating that the CS film had no scavenging ability on ROS.

Macrophages release numerous immune factors when they are activated by external stimulations and play vital roles in immune responses and host defense [51]. Nitric oxide (NO) and Interleukin 6 (IL-6) are primary molecules involved in macrophage-mediated innate immune responses and are commonly used to evaluate the immunomodulatory activity of compounds [52]. In the current study, LPS treatment caused a significant increase in NO and IL-6 production in RAW 264.7 cells. The result of NO secretion is shown in Figure 7C. The LPS-induced NO production could only be inhibited in the group of U/CS/T-GChl. There was no statistical difference between the other groups and the LPS group, indicating that the CS, U/CS/T, and U/CS/T-G films activated no host immune cells into inflammation-mediated cytotoxic conditions induced by NO generation. On the other hand, as shown in Figure 7D, LPS-induced IL-6 production was reduced by all the complex films but not the CS film. Moreover, the U/CS/T-GChl exhibited the best ability to reduce IL-6 secretion. The results are similar to the ability of films in scavenging ROS (Figure 7B). We speculated that the optimal immunomodulatory ability of the U/CS/T-GChl film might be related to the release of ulvan (Figure 2) and the addition of chlorophyll. Chlorophyll extracted from brown algae could inhibit NO production by inhibiting the expression of iNOS protein in LPS-induced RAW264.7 cells [53]. Phytol, the component of the side chain of chlorophyll, attenuated the induced inflammation in rat paw edema and reduced TNF-α and IL-6 production in the inflamed site [54]. On the other hand, ulvan has been reported to have immunomodulatory activity [55]. However, the immunomodulatory activities and effects of ulvan could vary widely even within the same species, and the structural characteristics of ulvan (such as molecular weight and extent of sulfation) also affected the results [56]. The determination of immune cells and cytokines is relatively simple, but the relationship between cytokines is complex, involving multiple interaction pathways [37]. Therefore, further research is needed for a deeper understanding.

### 3.8. In Vivo Wound Healing Activity

**Figure 8:**
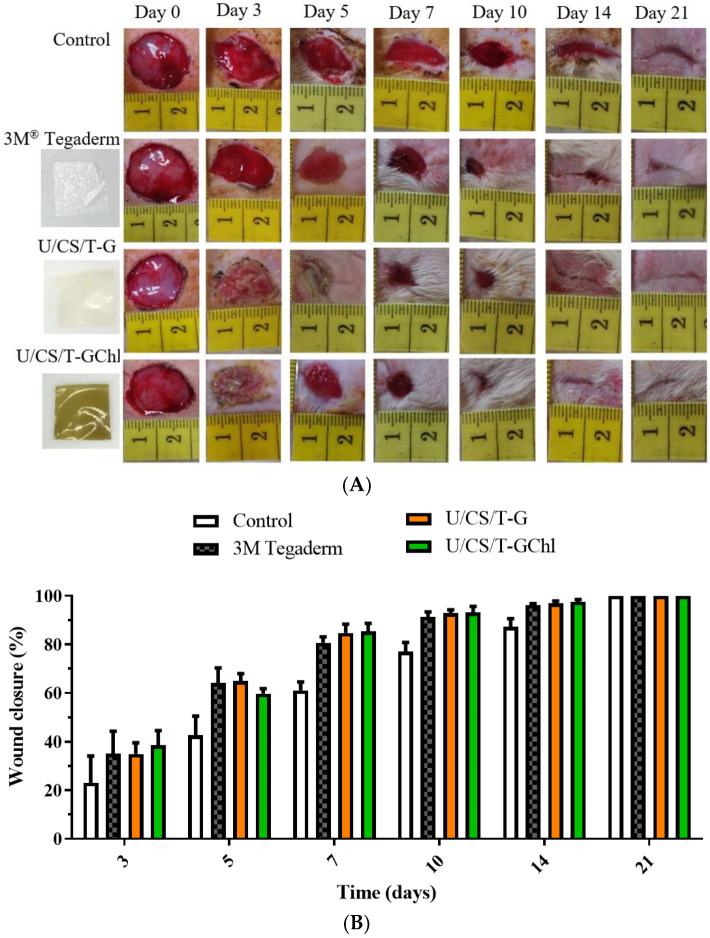
(**A**) Photographs of all skin layer wounds covered with a commercial dressing and the ulvan/chitosan complex films on SD rats at several specific days (0–21 days) after surgery. (**B**) Wound closure percentage of all skin layer wounds on SD rats at several specific days (3–21 days) after surgery. The control group is the open wound without any dressing.

To evaluate the wound-healing effect of the ulvan/chitosan complex films, the wound-healing assay in vivo was performed via a Sprague Dawley rat model. Figure 8A shows photographs of post-operative wound closure at several specific days; at the same time, a quantification of the wound closure (%) for each group was calculated and shown in Figure 8B. After 3 days post-surgery, we observed considerable exudate in the control and 3M^®^ Tegaderm group, resulting in a wettish wound. In the U/CS/T-G and U/CS/T-GChl groups, there was no soaking wet in the wound, and the wounds with a moderate moist environment were observed. The wound closure percentages (%) of the U/CS/T-G and U/CS/T-GChl films were already significantly higher than that of the control group. After 7 days post-surgery, both U/CS/T-G and U/CS/T-GChl films maintained wound moistness, with no accumulation of exudate and no signs of inflammation or infection. In contrast, the wounds in the control group continued to excrete exudate, prolonging the healing process. Compared with the control group, the U/CS/T-G and U/CS/T-GChl films exhibited higher percentages of wound closure, indicating the ability of U/CS/T-G and U/CS/T-GChl films to promote wound contractibility. Epithelialization can be accelerated by maintaining a moist wound environment and avoiding exudate accumulation [5]. The WVTRs of the complex films were close to 2000 g m^−2^day^−1^ (cf. Figure 1D), which was reported to be suitable for maintaining a moist wound surface, and thus, facilitating epidermal cell migration [57]. After 21 days post-surgery, the majority of the wound tissue was already repaired in all the groups, and there was no obvious residual scar tissue. According to the experimental results, the U/CS/T-G and U/CS/T-GChl films we developed had similar efficacy in wound healing to the commercially available 3M^®^ Tegaderm dressing.

**Figure 9:**
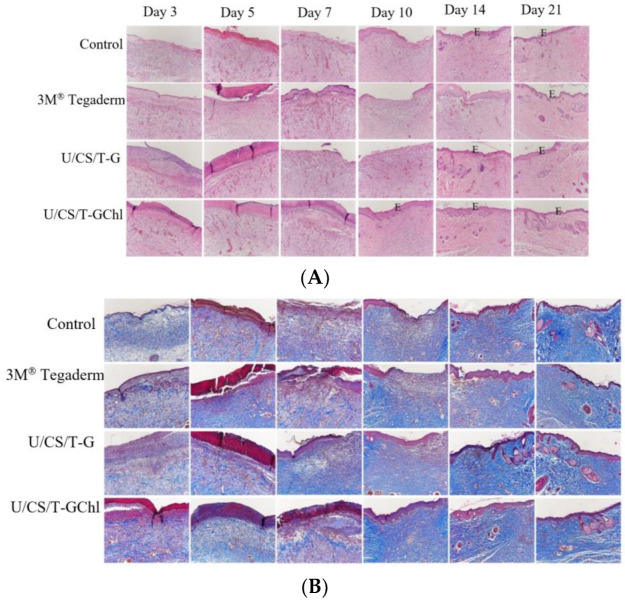
(**A**) Histological examination of skin wounds by H&E staining at several specific days (0–21 days) after surgery in SD rats. E: Epidermis. The control group is the open wound without any dressing. (100×) (**B**) Histological examination of collagen of skin wounds by Masson’s trichrome (MT) staining, 3 days up to 21 days in SD rats. The control group is the open wound without any dressing. (100×).

Histological analysis by H&E staining could elucidate the promoting effects of the ulvan/chitosan complex films on wound healing (Figure 9A). By day 5 post-surgery, wounds in all the groups were not yet epidermalized; however, more red blood cell accumulation was observed in the U/CS/T-G and U/CS/T-GChl groups. By day 10 post-surgery, we observed the complete re-epithelialization of wounds in the U/CS/T-GChl group, but not in the other three groups. The granulation tissue of the wound gradually thickened with time. By day 14 post-surgery, the U/CS/T-G and U/CS/T-GChl groups exhibited thicker granulation tissue and more extensive development of hair follicles and glands as compared with the control or 3M^®^ Tegaderm group. By day 21 post-surgery, the U/CS/T-GChl group had the most complete epithelialization, hair follicles, and glands in the wound area. Acceleration of wound contraction and re-epithelialization in the U/CS/T-G and U/CS/T-GChl groups indicated improvement in the overall healing process. Type I collagen is the main component in the extracellular matrix of skin tissue. Therefore, we used Masson’s trichrome (MT) staining to identify collagen [58] and determined the distribution of extracellular matrix in the regenerated tissues. The intensity of blue color in the tissue sections indicated the relative quantity of collagen. As shown in Figure 9B, the amount of secreted collagen increased with time in all the groups, as evidenced by the intensity of MT staining. However, we observed higher collagen content overall in the U/CS/T-G and U/CS/T-GChl groups when compared to the control or 3M^®^ Tegaderm group, suggesting that wounds treated with U/CS/T-G and U/CS/T-GChl films secreted more collagen.

**Figure 10:**
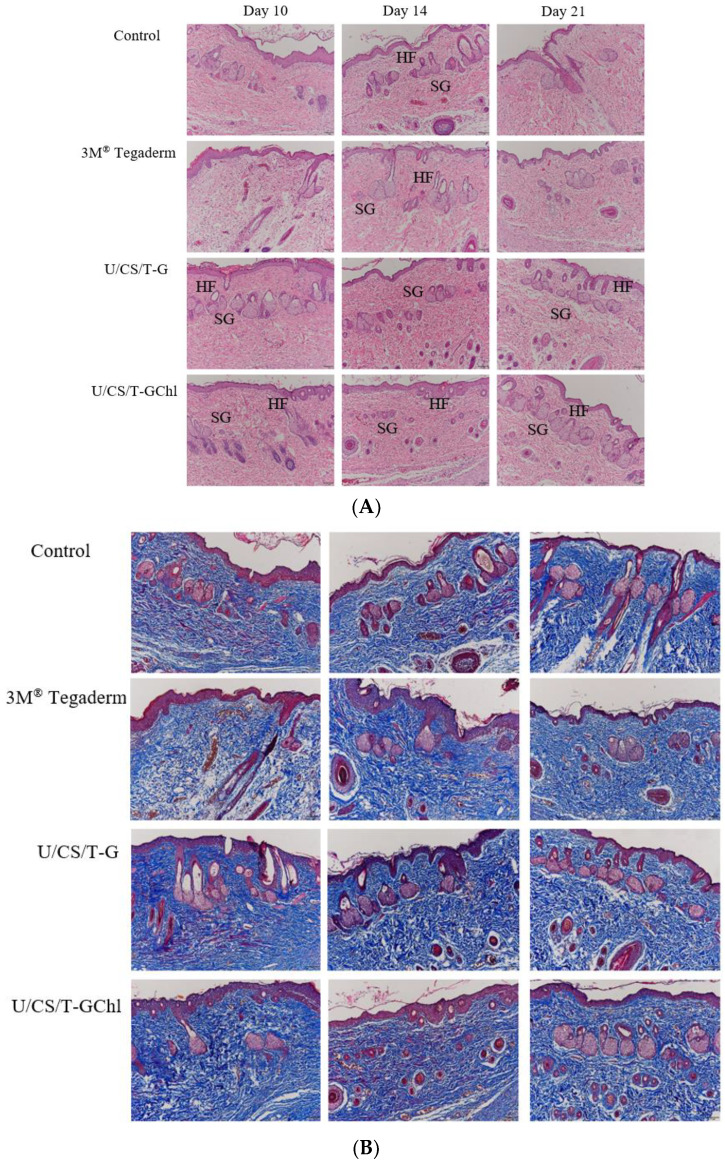
(**A**) Histological examination of skin wounds by H&E staining on day 10, 14, and 21 in SD rats. SGs: Sweat glands, HFs: hair follicles. The control group is the open wound without any dressing. (100×) (**B**) Histological examination of collagen in skin wounds by Masson’s trichrome (MT) staining on day 10, 14, and 21 in SD rats. The control group is the open wound without any dressing. (100×).

In order to clearly observe wound healing in rats, the repaired areas at the later stage of wound healing on day 10, 14, and 21 were re-examined by H&E staining and MT staining. The results of H&E staining are shown in Figure 10A. On day 10, we observed that the U/CS/T-G and U/CS/T-GChl groups formed a continuous smooth epidermal layer, and more new glands and hair follicles were also observed in the tissue. In contrast, the epidermis in the 3M^®^ Tegaderm group and the control group was less continuous and smooth. On day 14, the new glands formed in the U/CS/T-G and U/CS/T-GChl groups were gradually intact, while both of the 3M^®^ Tegaderm and control group had intact epidermis formation, but their subcutaneous tissue remained disorganized. Until day 21, we still observed that the U/CS/T-G and U/CS/T-GChl groups had a more complete tissue arrangement in the dermis than the other groups. The results of MT staining are shown in Figure 10B. Compared with the 3M^®^ Tegaderm and the control group, the U/CS/T-G and U/CS/T-GChl groups had darker blue and densely deposited collagen. In addition, there were more new glands in the subcutaneous tissue, and the arrangement was more complete. In brief, both U/CS/T-G and U/CS/T-GChl films exhibited good wound-repair ability.

## 4. Conclusions

Ulvan/chitosan complex films were prepared via the ionic crosslinking of chitosan through the addition of tripolyphosphate. In addition, glycerol and chlorophyll were added to enhance mechanical properties and bioactivities, respectively. The prepared complex films had moderate tensile strength and high elasticity. In addition, their water vapor transmission rates were in the range of 1791−2029 g/m^2^−day, and therefore, they could provide a suitable environment for wound healing. Most importantly, these complex films could release ulvan in a short period of time and their culturing media after release were non-toxic toward NIH 3T3 and HaCaT cells, and even promoted the migration of NIH 3T3 cells. These complex films also exhibited antioxidant activities which were ascribed not only to the films themselves but also to the released ulvan in the medium, and the addition of chlorophyll further increased antioxidant activities. Pretreatment with complex films before H_2_O_2_ exposure thus improved the cell viability of HaCaT in which the U/CS/T-GChl group was the most effective to prevent oxidative damage. This was ascribed to the synergistic effect of ulvan and chlorophyll. Moreover, these ulvan/chitosan complex films could reduce the production of reactive oxygen species (ROS); the addition of chlorophyll into the films effectively reduced the inflammatory response induced by LPS, evidenced by the reduction in NO and IL-6. From the result of the animal model, the U/CS/T-G and U/CS/T-GChl films could promote wound healing in the early stage and accelerate the regeneration of dermal glands and collagen production. These ulvan/chitosan complex films, which had good physiochemical properties, biological activity, and wound-repair ability, thus have potential to be applied as wound-dressing materials, particularly the one with the addition of chlorophyll.

## Data Availability

Not applicable.

## Supplementary Materials

The following supporting information can be downloaded at: https://www.mdpi.com/article/10.3390/polym14245382/s1, the details of the experimental methods on the antioxidant abilities. Figure S1: H_2_O_2_ induced cytotoxicity of HaCaT cells at different times by MTT assay. Control is the medium without H_2_O_2_. Figure S2: FTIR spectra of the U/CS/T film (20/75/5) and its individual components of chitosan (CS), ulvan (U), and tripolyphosphate (T).
