# Supplementary material for: In Situ Release of Ulvan from Crosslinked Ulvan/Chitosan Complex Films and Their Evaluation as Wound Dressings
Source: Polymers (Basel). 2022 Dec 8;14(24):5382. doi: 10.3390/polym14245382 (PMC9786826; doi:10.3390/polym14245382)
Supplement: Supplementary file 1 [file polymers-14-05382-s001.zip › polymers-2019112-supplementary.pdf]

Supplementary

In situ Release of Ulvan from Crosslinked Ulvan/Chitosan Complex Films and Their Evaluation as Wound Dressings

Trong-Ming Don [1,*], Chen-Han Ma [2] and Yi-Cheng Huang [2,*]

Supplementary Experimental

Anti-oxidant activities

1,1-diphenyl-2-picrylhydrazyl (DPPH) scavenging activity

Samples (250 μL) were mixed vigorously with 250 μL 0.2 mM DPPH methanolic solution and left to stand for 30 min at room temperature in the dark. The absorbance was measured at 517 nm using a SpectraMax 340PC384 microplate spectrophotometer. Sample substituted with ddH_2O was used a control group. The DPPH scavenging activity (%) was determined using the following equation:

$$\text{Scavenging activity (\%)} = \left(1 - \frac{OD_{517\ \text{Sample}}}{OD_{517\ \text{Control}}}\right) \times 100\%$$

Superoxide radical scavenging activity

Various solutions of β-nicotinamide adenine dinucleotide (NADH, 78 μM), nitroblue tetrazolium (NBT, 50 μM) and phenazinmethosulfate (PMS, 10 μM) were prepared in the tris-HCl buffer (16 mM, pH 8.0). 200 μL of sample solution was added sequentially with NBT, NADH and PMS solutions, each 200 μL. After incubating the solution for 15 min at room temperature in the dark, the absorbance was measured at 560 nm using a SpectraMax 340PC384 microplate spectrophotometer. In the control, sample was substituted with ddH_2O. The scavenging activity (%) was calculated by the equation given below:

$$\text{Scavenging activity (\%)} = \left(1 - \frac{OD_{560\ \text{Sample}}}{OD_{560\ \text{Control}}}\right) \times 100\%$$

Ferrous ion chelating activity

The reaction mixture, containing 250 μL of sample, 5 μL of $FeCl_2$ (2 mM) and 10 μL of

ferrozine (5 mM), were mixed thoroughly and allowed to react for 10 min in the dark. The absorbance was measured at 562 nm using a SpectraMax 340PC384 microplate spectrophotometer. In the control, sample was substituted with ddH_2O. The chelating activity (%) was calculated by the equation given below:

$$\text{Chelating activity (\%)} = \left(1 - \frac{OD_{562\ \text{Sample}}}{OD_{562\ \text{Control}}}\right) \times\ 100\%$$

Reducing power

Samples (250 μL) were prepared in PBS (0.2 M, pH 6.6) and added with 250 μL potassium ferricyanide (1%, w/v); they were then incubated at 50 °C for 20 min. After terminating the reaction by addition of 500 μL trichloroacetic acid solution (10%, w/v), the solution was mixed with 300 μL ferric chloride (0.1%, w/v). Finally, the absorbance was measured using a SpectraMax 340PC384 microplate spectrophotometer at 700 nm.

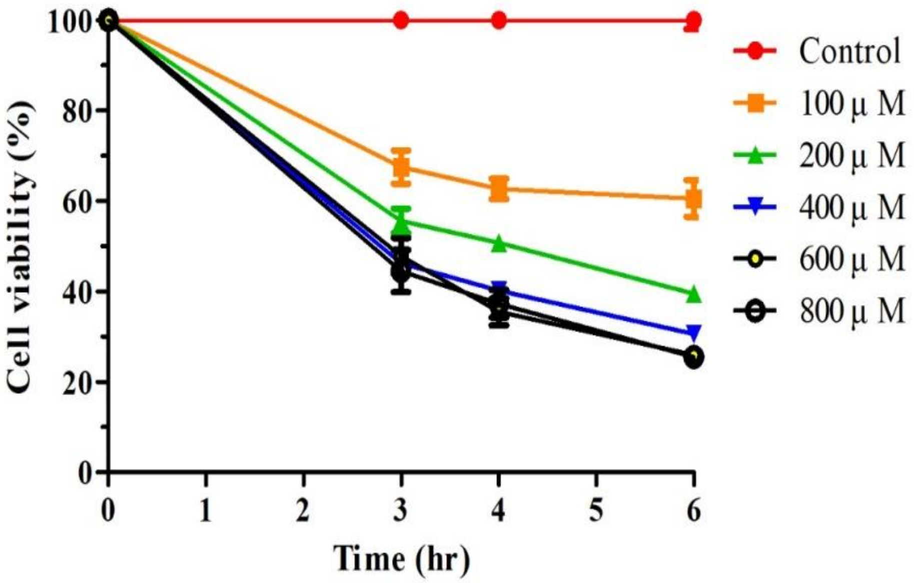

Figure S1. H_2O_2 induced cytotoxicity of HaCaT cells at different times by MTT assay. Control is the medium without H_2O_2.

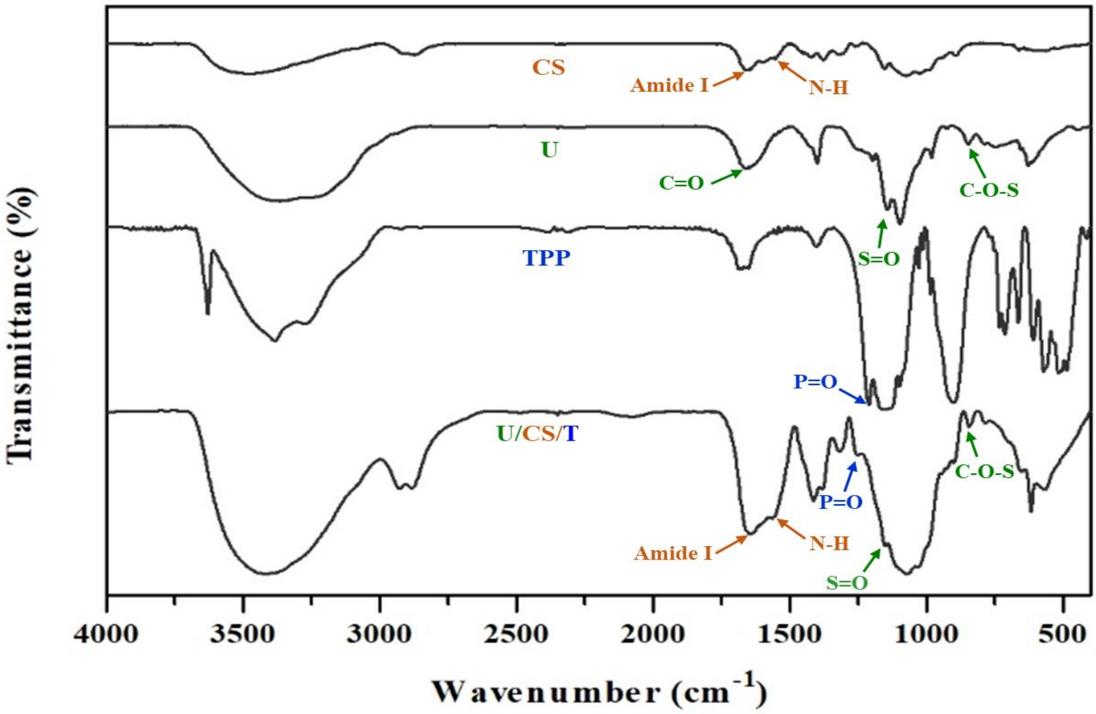

Figure S2. FTIR spectra of the U/CS/T film (20/75/5) and its individual components of chitosan (CS), ulvan (U), nd tripolyphosphate (T).
